# The COVID-19 pandemic’s intersectional impact on work life, home life and wellbeing: an exploratory mixed-methods analysis of Georgia women’s experiences during the pandemic

**DOI:** 10.1186/s12889-022-14285-4

**Published:** 2022-10-31

**Authors:** Megan McCool-Myers, Damion Grasso, Debra Kozlowski, Sarah Cordes, Valerie Jean, Heather Gold, Peggy Goedken

**Affiliations:** 1 Department of Gynecology and Obstetrics, Emory University School of Medicine, 46 Armstrong St SE, 30303 Atlanta, GA USA; 2 Department of Psychiatry, University of Connecticut Health, Connecticut, USA

**Keywords:** COVID-19, Latent class analysis, Mixed methods, Adverse experience, Exposure, Female, Mental health, Intimate partner violence, Stress

## Abstract

**Background:**

Women have been especially impacted by the COVID-19 pandemic. This exploratory study aimed to characterize women’s adverse experiences related to their work, home lives, and wellbeing during the height of the COVID-19 pandemic and to describe demographic differences of those lived experiences.

**Methods:**

Using the validated Epidemic-Pandemic Impacts Inventory, we collected data from reproductive-aged women in the state of Georgia about their exposure to adverse events during the pandemic. A latent class analysis (LCA) was performed to identify subgroups of women reporting similar adverse experiences and describe their sociodemographic characteristics. An optional open-ended question yielded qualitative data that were analyzed thematically and merged with subgroup findings. Data were collected from September 2020 to January 2021.

**Results:**

423 individuals aged 18–49 completed the survey with 314 (74.2%) providing qualitative responses. The LCA yielded 4 subgroups: (1) a “low exposure” subgroup (n = 123, 29.1%) with relatively low probability of adverse experiences across domains (e.g. financial insecurity, health challenges, barriers to access to healthcare, intimate partner violence (IPV)); (2) a “high exposure” subgroup (n = 46, 10.9%) with high probability of experiencing multiple adversities across domains including the loss of loved ones to COVID-19; (3) a “caregiving stress” subgroup (n = 104, 24.6%) with high probability of experiencing challenges with home and work life including increased partner conflict; and (4) a “mental health changes” subgroup (n = 150, 35.5%) characterized by relatively low probability of adverse experiences but high probability of negative changes in mental health and lifestyle. Individuals in subgroups 1 and 4, which had low probabilities of adverse experiences, were significantly more likely to be non-Hispanic white. Individuals in subgroup 2 were more likely to identify with a sexual or racial/ethnic minority population. Inductive coding of qualitative data yielded themes such as stress, mental health, financial impact, and adaptation/resilience, providing context for pandemic-related adversity.

**Conclusion:**

Though many individuals in our sample experienced hardship, minority populations were unequally impacted by pandemic-related adversity in work life, home life, and wellbeing. Recovery and future emergency preparedness efforts in Georgia must incorporate support mechanisms for mental health and IPV, focusing especially on the intersectional needs of racial, ethnic, and sexual minorities.

**Supplementary information:**

The online version contains supplementary material available at 10.1186/s12889-022-14285-4.

## Background

At the start of 2020 a novel coronavirus, causing an infectious respiratory disease SARS-CoV-2 (COVID-19), spread globally. As of December 2021, the US had the highest number of reported cases and deaths of any country worldwide (50 million cases, 797,348 deaths). [1] Southeastern US states have been particularly impacted: the state of Georgia has lost nearly 30,000 lives during the pandemic and is ranked 7th nationwide in the rate of deaths per 100,000. [1, 2].

To reduce the spread of infection in early 2020, restrictive measures such as school closures, business closures, and reduced social interaction were implemented. These measures significantly impacted the lives of American families, [3] with women experiencing disproportionate effects on their work life, home life, and wellbeing. Job loss rates in Spring 2020 were nearly two times higher for women than men. [4] When schools closed, women took on many additional responsibilities, including even more childcare than usual, assisting with remote learning, and in some cases shifting entirely to homeschooling. [4] With couples and families obligated to stay at home, in many cases with reduced or no work, these existential insecurities contributed to increased intimate partner violence (IPV) towards women. [5, 6] These factors and others have negatively impacted women’s mental health: in April 2020 rates of depression and anxiety in women were two to three times higher than before the pandemic. [7].

The purpose of this study is to describe Georgia women’s pandemic-related adverse experiences in work life, home life and wellbeing through a quantitative and qualitative approach. Our mixed-methods strategy will first identify subgroups of women with shared experiences by way of a latent class analysis and then enrich these findings with qualitative insights from women in each subgroup. Characterizing women’s experiences in Georgia could inform the development of health services or public health initiatives to support women and their families during the continuing COVID-19 pandemic.

## Methods

### Sample

To answer our research question, we conducted a cross-sectional, online survey. Inclusion criteria were: age 18–49, assigned female at birth, and living in Georgia since January 2020. Two million women ages 18–49 live in the state; [8] we selected a sample size of 400 to yield standard errors of ±5%. Emory University Institutional Review Board approved this study on May 19, 2020 (MOD003-STUDY00000657).

### Procedures

We employed a mixed-methods approach, using data collected through an online survey, to characterize women’s adverse outcomes experienced during the COVID-19 pandemic. Data were collected from September 2020 through January 2021. We recruited individuals through two sources: a state-wide network for research volunteers (study invitations sent via email) and social media (respondents targeted through paid advertisements on Facebook, see Additional file 1 for sample advertisement). We used quota sampling to attain similar distributions of demographic characteristics as reported in state estimates; the study team monitored the demographic data of the participants (specifically age group, race, and setting) to prevent oversampling. We dynamically adapted the screener, recruitment strategy, and advertising to address gaps in sampling. We followed best practice guidelines for reporting results. [9].

Individuals interested in the study were directed to the online survey, administered through a secure web-based application REDCap. The survey was pilot tested with n = 5 individuals prior to launch and revised accordingly. Informed consent was obtained from all subjects. After completing the screener, they completed the survey questions. The survey entailed multiple-choice and open-ended questions to capture both quantitative and qualitative data. We explored the following domains through the use of validated items or items from peer-reviewed publications: sociodemographic and economic data, work life, home life, emotional/ physical/mental wellbeing, and COVID-19 infection. Multiple-choice questions for the quantitative analysis were drawn primarily from the Epidemic-Pandemic Impacts Inventory from Grasso et al. [10] Given the increase in intimate partner violence (IPV) during the pandemic, it was important to incorporate questions about current IPV and lifetime IPV in this survey. [5, 6, 11] The survey concluded with an open-ended item: “Please describe the impact that the COVID-19 pandemic has had on you and your family,” which was used for the qualitative analysis. At conclusion, survey respondents provided their email addresses and were sent an electronic $5 gift card as compensation. Resources for domestic violence, substance use, mental health, suicide prevention and family planning were listed at the end of the survey. The final survey can be found in Additional file 2.

### Mixed methods analysis

First, we calculated frequencies and percentages for categorical variables. Multi-category variables were dichotomized to facilitate the quantitative analysis.

Next, we performed a latent class analysis (LCA) to identify different subgroups within populations that share certain outward characteristics. [12, 13] Subgroups are referred to as latent groups (or classes). [12] Each individual in a sample can only belong to one class. The LCA was applied in several steps: indicators were entered into the LCA beginning with one class and adding classes incrementally until a unique solution could not be determined with maximum likelihood (ML) methods. Several fit indices were examined and used to determine optimal fit. Information criterion indices include the Bayesian Information Criteria (BIC), [14] Sample Size Adjusted Bayesian Information Criterion, [15] Consistent Akaike Information Criterion, [16] and Approximate Weight of Evidence, [17] which are interpreted such that lower values convey better fit. BIC is the most commonly used and relied upon fit index for comparing models. [18, 19] Additionally, the Vuong-Lo-Mendell-Rubin Likelihood Ratio Test [20] and the Bootstrap Likelihood Ratio Test [21] were applied to compare between models; nonsignificant values indicate the model with one additional class is not a statistically improved fit over the current model. The Bayes Factor (BF) [22, 23] is interpreted such that a BF of < 3 is considered weak evidence that the model with one fewer class is superior over the model with one additional class, BF of 3–10 conveys moderate evidence, and BF > 10 conveys strong evidence for the model with one fewer class. The approximate correct model probability provides an estimate of the probability that a given model is “correct” among the set of tested models under the assumption that one of the models is “correct”. [14] Entropy values were used to evaluate the quality of classes and ranged from 0 to 1, with values closer to 1 representing better separation of classes. [24] Univariate entropy scores were examined to evaluate the relative contribution of individual items in separating classes.

Then, to examine associations between classes and sociodemographic characteristics, we used the Mplus DU3STEP procedure described by Lanza et al. [25] These procedures follow 3 steps: (1) the LCA is estimated without covariates or distal outcomes, (2) the highest probability of class membership is used to assign classes, and (3) associations between class membership and outcomes are estimated with an adjustment based on classification uncertainty. These methods perform well when class separation is sufficient (i.e., entropy > 0.60). Alpha was adjusted for pairwise comparisons using the Bonferroni procedure. [26].

Finally, two researchers thematically analyzed the responses to the open-ended question on women’s experiences during the pandemic. Statements describing mental health problems, additional responsibilities, stress, financial issues, social isolation and/or other pandemic-related challenges were classified as “negative”; statements describing “breaking even” or “counting one’s blessings” were considered “neutral”; statements describing improvements were deemed “positive”. In a second analysis, inductive themes were iteratively extracted from the responses to create a codebook; [27] multiple themes could be allocated to each response. After independent assessment, the researchers met to discuss and resolve coding discrepancies. We selected anchor quotes to expound upon the experiences of members of each class. We used a Chi-squared test to determine if there was a significant difference in the proportion that responded to this question across the classes.

## Results

### Sample characteristics

Of the 436 respondents who started our online survey, 13 screened out or did not complete the survey, yielding a final sample of 423 (97.0% completion rate). [28] Average completion time was 14 min (median 12 min., range 4–59 min.). Table 1 provides sociodemographic and economic characteristics of the 423 respondents. Our sample was generally younger, more educated, and lower income than the state population. The proportion of respondents identifying as heterosexual was significantly lower (83.7%) than population estimates for GA (95.1%). See Additional file 3 for further comparisons between sample characteristics and state estimates.

**Table 1:** Sociodemographic and economic characteristics of survey respondents, Georgia US, 2020 (N = 423)

Age (years)	
18–25	81 (19.1)
26–30	79 (18.7)
31–35	110 (26.0)
36–40	63 (14.9)
41–45	63 (14.9)
46–49	27 (6.4)
Race	
White	249 (58.9)
Black	125 (29.6)
Asian	29 (6.9)
Other or multiple races	17 (4.0)
Decline to answer	3 (0.7)
Ethnicity	
Non-Hispanic	385 (91.0)
Hispanic	32 (7.6)
Decline to answer	6 (1.4)
Gender	
Female	420 (99.3)
Non-Binary	3 (0.7)
Sexual orientation	
Heterosexual	343 (81.1)
Bisexual	52 (12.3)
Homosexual	15 (3.5)
Other	12 (2.8)
Decline to answer	1 (0.2)
Education level	
High school/GED	58 (13.7)
Associates degree/some college	125 (29.6)
Bachelor’s degree	122 (28.8)
Graduate degree	117 (27.7)
Decline to answer	1 (0.2)
Relationship status	
Partnered or married	268 (63.4)
Single, separated, divorced	154 (36.4)
Decline to answer	1 (0.2)
Residential setting	
Urban	142 (33.6)
Suburban	197 (46.6)
Rural	84 (19.9)
Children living in home	
No	212 (50.1)
Yes	211 (49.9)
Income level	
High	139 (32.9)
Middle	118 (27.9)
Low	117 (27.7)
Don’t know/decline to answer	49 (11.5)
Monthly household income	
Has decreased	189 (44.7)
Has remained the same	175 (41.4)
Has increased	53 (12.5)
Decline to answer	6 (1.4)
Health insurance	
Currently have health insurance	353 (83.5)
Lost health insurance due to COVID-19	20 (4.7)
Lost health insurance, other reason	46 (10.9)
Decline to answer	4 (0.9)

### Quantitative analysis

The COVID-19 pandemic impacted respondents across all domains: work life, home life, wellbeing, COVID-19 infection, and partner abuse (Table 2). 22% had experienced job loss (n = 93). Nearly one-third (n = 132, 31.2%) stated that they were unable to pay bills; 1 in 5 households (n = 85, 20.1%) reported food shortages. Of the respondents with children, 75.8% (n = 160/211) had to take over teaching their children from home. Most respondents reported increases in mental health issues and sleep problems; physical health problems and substance use reportedly increased. Thirty individuals in our sample (7.1%) had tested positive for COVID-19. Every 5th person had lost a family member or close friend to COVID-19 infection (n = 75, 17.8%). Twenty-one individuals (5.0%) were currently exposed to intimate partner violence (IPV).

**Table 2:** Frequencies of responses to questions about COVID-19 impact on work life, home life, wellbeing, infection and partner abuse, Georgia US, 2020 (n = 423) ^a^

	N (%)
Work life (n = 423)	
Laid off	93 (22.0)
Reduced work hours	163 (38.5)
Continued working despite exposure	194 (45.9)
Hard time working from home	146 (34.5)
Hard time working due to caretaking	146 (34.5)
Difficulty with transportation	93 (22.0)
Unable to pay bills	132 (31.2)
Difficulty getting food or healthy food	85 (20.1)
Home life (n = 211)*	
Having to take over teaching children	160 (75.8)
Childcare unavailable	108 (51.2)
Difficulty taking care of children	105 (49.8)
Increased conflict with children	77 (36.5)
Increased conflict among children	77(36.5)
Increased conflict with adults**	105 (24.8)
Wellbeing (n = 423)	
Increased physical health problems	125 (29.6)
Increased mental health problems	327 (77.3)
Increased sleep problems	299 (70.7)
Increased alcohol/substance use	119 (28.1)
Less physical activity	283 (66.9)
Overeating	276 (65.2)
Barriers to healthcare	60 (14.2)
Barriers to mental health services	86 (20.3)
Less routine healthcare	216 (51.1)
Unable to get medications	61 (14.4)
COVID-19 infection (n = 423)	
Tested positive for COVID-19	30 (7.1)
Had COVID-19 symptoms but never tested	40 (9.5)
Provided direct care to COVID-19+	46 (10.9)
Provided supportive care to COVID-19+	55 (13.0)
Death of family/friend due to COVID-19	75 (17.8)
Partner abuse (n = 423)	
Lifetime intimate partner violence	106 (25.1)
Current intimate partner violence	21 (5.0)

^a^ Based on a population of N = 423 individuals assigned female at birth; n = 3 identify as non-binary while the remainder identify as female

* Denominator of n = 211 representing those respondents who reported having children

** Denominator of N = 423, including all respondents with or without children

Frequencies of responses were analyzed further in a latent class analysis (LCA). Results of the LCA revealed a potential fit of a 2-class, 4-class, and 5-class model, given the superior values of various indices (Table 3). However, the 4-class model was selected as the optimal model as it had the lowest Bayesian Information Criterion (BIC). The 4-class model had the highest BF at 3.244, conveying moderate evidence that this model is the most appropriate fit for the data. Although the 2-class model had a slightly higher entropy score, the 4-class model suggests a good separation of classes.

**Table 3:** Fit statistics and classification coefficients for latent class analysis ^a^

	d	LL	BIC	SABIC	CAIC	AWE	BLRT *p*	VLMR-LRT *p*		*BF*		Ent-ropy		*cmP*	
1 Class	32	-6914.01	14021.54	13919.99	13944.06	13960.06	-		-		0.000		-	0.000	
2 Classes	65	-6285.78	12964.64	12758.37	12807.27	12839.77	< 0.001		< 0.001		0.000		**0.899**	0.000	
3 Classes	98	-6095.32	12783.29	12472.30	12546.02	12595.02	< 0.001		**0.008**		0.005		0.884	0.049	
4 Classes	131	-5943.47	**12678.68**	12262.97	12361.99	12427.49	< 0.001		0.148		**3.244**		**0.887**	0.455	
5 Classes	164	**-5855.23**	12702.22	**12181.79**	**12305.18**	**12387.18**	< 0.001		0.577		-		0.885	**0.484**	
6 Classes	197	-	-	**-**	**-**	**-**	-		-		-		-	-	
7 Classes	230	-	-	-	**-**	**-**	**-**		-		-		-	**-**	

*Note*. d = number of parameters; LL = log-likelihood; BIC = Bayesian Information Criterion; SABIC = Sample size adjusted BIC; CAIC = Consistent Akaike Information Criterion; AWE = Approximate Weight of Evidence Criterion; VLRM-LRT = Vuong-Lo-Mendell-Rubin adjusted likelihood ratio test; *p* = *p-value; BF* = Bayes Factor; *cmP* = correct model probability. Bold values indicate superior fit for each statistic

^a^ Based on a population of N = 423 individuals assigned female at birth; n = 3 identify as non-binary while the remainder identify as female

**Fig. 1:**
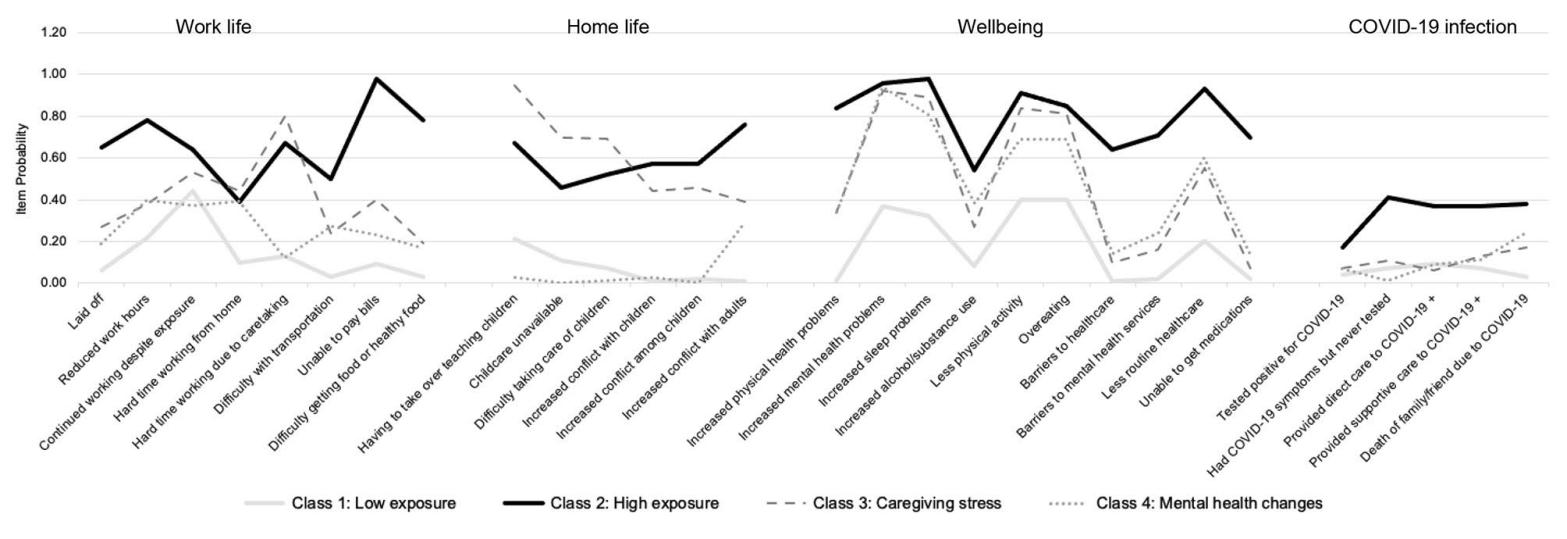
**Probability of adverse experiences during the COVID-19 pandemic (N = 423 respondents assigned female at birth)**. Probabilities of adverse experiences surrounding work life, home life, wellbeing and COVID-19 infection were generally lowest for Class 1 and highest for Class 2. Members of Class 3 had high probabilities of reporting challenges in caring for family members, while women in Class 4 reported negative experiences related to their mental and/or physical wellbeing

Conditional item probabilities and class differences on sociodemographic and economic variables were used to further characterize and label the four classes (see Additional file 4). We labeled these classes as follows: Class 1 “Low exposure” to adverse experiences, Class 2 “High exposure”, Class 3 “Caregiving stress,” and Class 4 “Mental health changes.” Fig. 1 illustrates the likelihood of reporting adverse pandemic-related experiences surrounding work life, home life, wellbeing, and COVID-19 infection for each of the classes. Table 4 shows the differences in sociodemographic characteristics for the four classes.

### Class 1 Low exposure

Women in Class 1 (n = 123, 29.1%) had a relatively low probability of reporting adverse experiences related to work life, home life, wellbeing, or COVID-19 infection. These women were generally older (30–49 years old), non-Hispanic white, and did not have children living in their home. Class 1 was most likely to have had a stable or increased household income during the pandemic. These women were least likely to have experienced IPV in their lifetimes.

### Class 2 High exposure

Individuals in Class 2 (n = 46, 10.9%) were likely to have experienced multiple adverse outcomes during the pandemic. They had the highest likelihood of reporting an inability to pay bills, increased conflict in the home, poor mental and physical health, and barriers to accessing health care. Furthermore, individuals in this class had the greatest probability of having had close or direct coronavirus exposure and of having lost a family member or friend due to COVID-19. Examining the demographics of members of Class 2, they were more likely to identify with a sexual minority or a racial/ethnic minority population; two of the three non-binary-identifying persons in the sample were placed in Class 2 based on their responses. People in Class 2 were least likely to have health insurance currently, they reported a lower household income since the pandemic, and they experienced IPV during the pandemic.

### Class 3 Caregiving stress

Members of Class 3 (n = 104, 24.6%) reported difficulties related to childcare, teaching children, and balancing work and caretaking. These women also experienced poor mental and physical wellbeing during the pandemic. Demographically, Class 3 was older (30–49) and non-white. Similar to Class 2, these women reported experiencing IPV during the pandemic.

### Class 4 Mental health changes

Individuals in Class 4 represented the largest group (n = 150, 35.5%). This subgroup had relatively low probability of reporting adverse experiences in work/home life or with COVID-19 infection, yet they did report increased mental health challenges and unhealthy lifestyle changes. Members of Class 4 were least likely to be married/partnered, and they were more likely to identify with a sexual minority population. One of the three non-binary-identifying persons was a member of Class 4 based on their responses.

**Table 4:** Class Comparisons

Sociodemographic Characteristics ^a^	Class 1 Low Exposure		Class 2 High Exposure		Class 3 Caregiving Stress		Class 4 Mental Health			Class Differences ^b^
	Prob	SE	Prob	SE	Prob	SE	Prob	SE	χ^*2*^	
Age group										
18–30	0.40	0.06	0.32	0.09	0.15	0.04	0.55	0.06		
31–49	0.60	0.06	0.69	0.09	0.85	0.04	0.45	0.06	32.96**	C3 > C1,C4; C2 > C4
Race/Ethnicity										
Non-Hispanic White	0.61	0.05	0.34	0.08	0.44	0.05	0.69	0.05		
Non-White	0.40	0.05	0.67	0.08	0.56	0.05	0.41	0.05	14.56*	C2,C3 > C1,C4
Setting										
Urban/Suburban	0.75	0.06	0.73	0.09	0.76	0.05	0.89	0.03		
Rural	0.25	0.06	0.27	0.09	0.24	0.05	0.11	0.03	9.66*	C1,C3 > C4
Children in Home										
No	0.80	0.06	0.00	0.00	0.00	0.00	0.95	0.03		
Yes	0.20	0.06	1.00	0.00	1.00	0.00	0.05	0.03	1621.27**	C2,C3 > C1,C4; C1 > C4
Insurance										
No	0.13	0.05	0.41	0.08	0.07	0.03	0.16	0.04		
Yes	0.87	0.05	0.59	0.08	0.93	0.03	0.87	0.04	17.27*	C1,C3,C4 > C2
Sexual orientation										
Heterosexual	0.91	0.03	0.54	0.09	0.93	0.03	0.73	0.05		
Bi/homosexual/other	0.09	0.03	0.46	0.09	0.07	0.03	0.27	0.05	23.16**	C2,C4 > C1,C3
Relationship status										
Single/separated	0.34	0.05	0.29	0.10	0.24	0.05	0.50	0.05		
Partnered/married	0.66	0.05	0.71	0.10	0.76	0.05	0.50	0.05	13.37*	C1,C2,C3 > C4
Highest education										
No graduate degree	0.38	0.21	0.65	0.09	0.49	0.51	0.36	0.06		
≥ Bachelor’s degree	0.62	0.21	0.35	0.09	0.51	0.11	0.64	0.06	7.61	*ns*
Income										
Low	0.25	0.07	0.49	0.09	0.31	0.07	0.30	0.06		
Middle/High	0.75	0.07	0.51	0.09	0.69	0.07	0.70	0.06	4.74	*ns*
Income change										
Lower than before	0.24	0.06	0.81	0.07	0.54	0.06	0.46	0.07		
Same or higher	0.76	0.06	0.19	0.07	0.46	0.06	0.55	0.07	47.79**	C1 > C2,C3,C4; C3,C4 > C2
Current IPV										
No	1.00	0.00	0.88	0.05	0.88	0.04	0.98	0.01		
Yes	0.00	0.00	0.12	0.05	0.12	0.04	0.02	0.01	22.18**	C2,C3 > C1,C4
Lifetime IPV										
No	0.94	0.03	0.59	0.09	0.68	0.06	0.68	0.05		
Yes	0.06	0.03	0.41	0.09	0.32	0.06	0.33	0.05	45.57**	C2,C3,C4 > C1

*IPV = intimate partner violence, C1 = Class 1, C2 = Class 2, C3 = Class 3, C4 = Class 4*

*Note.* Class differences represent pairwise comparisons that are significant after Bonferroni adjustment

*p < .01, **p < .001

^a^ Class comparisons are illustrated with the class(es) showing the highest probability for a particular variable appearing on the left side of the ‘greater-than’ sign

^b^ Based on a population of N = 423 individuals assigned female at birth; n = 3 identify as non-binary while the remainder identify as female

### Qualitative analysis

**Fig. 2:**
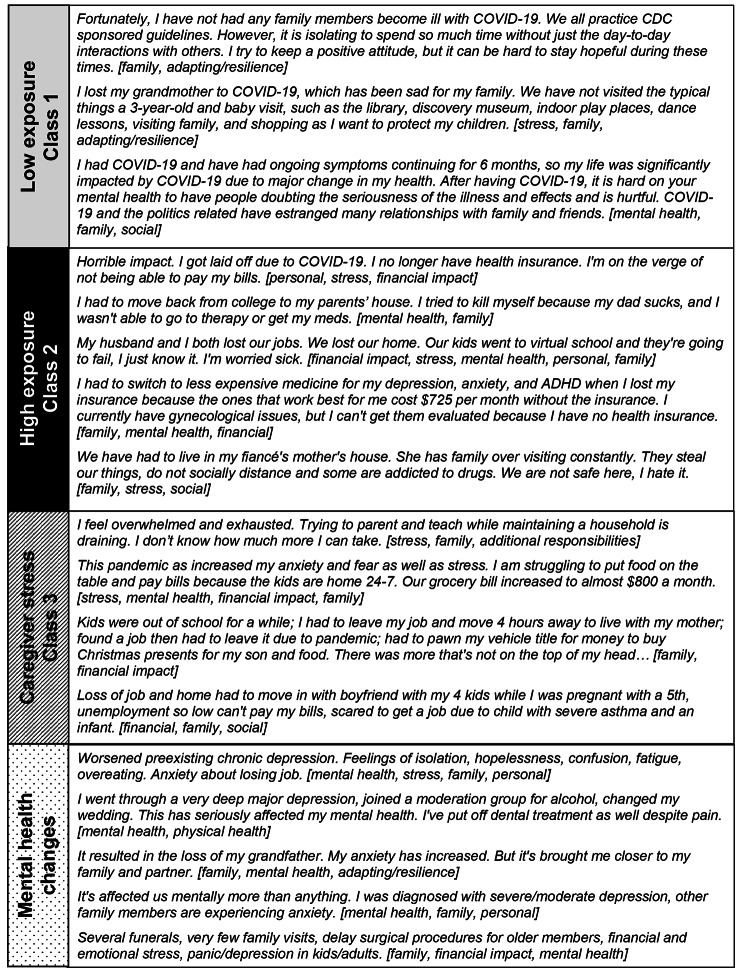
**Selected quotes describing pandemic-related adverse events (n = 309 respondents assigned female at birth)**. Selected anchor quotes that illustrate a commonality of themes - stress, family, mental health, financial impact, and adaption/resilience - and simultaneously the distinct experiences among individuals in each class. Inductive themes identified through thematic analysis are presented in brackets

Participants were invited to expand on the pandemic’s impact on their lives in an optional open-ended question. In total 330 individuals responded to the question. We excluded 21 responses that were deemed invalid or illogical by the researchers, leaving 309 valid responses (73.0%). There was no significant difference between the proportion of classes that responded / did not respond to the question. Responses were primarily negative (n = 264, 85.4%), followed by some that were neutral (n = 36, 11.7%) or positive (n = 9, 2.9%). Predominant themes which emerged from the negative quotes were stress, family, mental health, financial impact, and adaptation/resilience. Figure 2 provides anchor quotes from individuals in each of the 4 classes and the identified themes.

## Discussion

Participants in our study were significantly impacted by the COVID-19 pandemic. People who experienced multiple adversities (Class 2 High exposure) were significantly more likely to identify as Black and/or Hispanic, illustrating the pandemic’s disproportionate impact on minority families. Individuals in Class 2 and Class 3 (Caregiving stress) had higher probabilities of experiencing IPV during the pandemic. Class 4 Mental health changes, the largest subgroup in our sample, was generally non-Hispanic white; for this group, the pandemic exacerbated feelings of anxiety, isolation, panic, and depression. Nearly 30% of our sample had a low probability of reporting adverse pandemic-related experiences (Class 1 Low exposure); some reported the same or even higher income than before the pandemic. Nonetheless, these women described family deaths, long-term COVID-19 symptoms, and damaged family relationships due to the pandemic.

Mental health issues were reported by more than 75% of individuals in our sample. Early data from the pandemic (March to June 2020) found that identifying as female is significantly associated with higher self-reported levels of stress, anxiety, depression, and posttraumatic stress symptoms, and more severe overall psychological impact of the pandemic. [29, 30] Furthermore, women who experience intimate partner violence are at high risk for developing mental health problems. [31] Lifetime prevalence of IPV in our sample was 25%, comparable with pre-pandemic estimates for the US. [32] Numerous studies and reports attest to the “horrifying surge” in domestic violence across the globe. [31, 33] 5% of our sample reported current IPV, and women who experienced abuse in the past are at increased risk of violence during the ongoing pandemic. [34] Given the stigma around mental health problems and the underreporting of domestic abuse, our data may not fully capture all adverse experiences of individuals in our sample. It is therefore of utmost importance that governments acknowledge the unique vulnerability of women and take action to improve conditions for this population during the pandemic and beyond. [33].

The COVID-19 pandemic heavily impacted the families of respondents with unequal impact among individuals in Class 2. For all surveyed, income loss was reported by 44.7% and job loss by 22.0%. This corresponds with a national survey of US women in which 48.0% reported income loss and 22.9% job loss. [35] These financial problems had implications for paying bills, buying food, and paying for healthcare. Members of Class 2 (High exposure) had the highest likelihood of reporting an inability to pay bills, food shortages, increased conflict in the home, poor mental and physical health, health insurance loss, and barriers to accessing health care. Tragically, individuals in Class 2 – who were the most impacted by the pandemic – were also most likely to have lost loved ones due to COVID-19. Qualitative input from individuals in Class 2 contextualized the severity of their personal experiences. For example, while “family” and “stress” may have emerged as themes in all four classes, the strains posed on those in Class 2 describe specific, and in some cases, multiple adverse experiences such as homelessness/home insecurity, suicidality, desperation, and food insecurity.

Our quantitative and qualitative research substantiate the disparate experiences of minority populations compared to white, straight populations during the pandemic. Racial, ethnic and sexual minority populations in the US are at increased risk of severe COVID-19 disease; [36, 37] in addition, these populations carry a disproportionate burden of social, economic and health challenges which have been magnified through the pandemic. [3, 6, 38] Minority populations in our sample were more likely to have serious, adverse experiences across multiple domains during COVID-19 than non-Hispanic white women and straight women. Conversely, Class 1, which reported fewest negative experiences, was generally non-Hispanic white. These women had middle to high income prior to the pandemic, and stable or even increased income during the pandemic; they reported no IPV, very few personal losses to COVID-19, and limited barriers to the care that they needed. The findings illustrate how intersections of multiple structures of inequalities (such as ethnicity and race) can have a multiplying effect when disadvantaged positions intersect in the same individual. [39] Future pandemic-related research must incorporate the lens of intersectionality and social determinants of health in order to design appropriate policy responses that mitigate, instead of increase, the potential unequal effect of this pandemic. [39].

### Limitations

Findings from this study add to the body of literature on the impacts of the COVID-19 pandemic and associated sociodemographic disparities. However, limitations inherent to this study were, first, the use of internet-based recruitment, which may not be accessible to all Georgians. Second, non-probability sampling limits the generalizability of our findings. Third, our sample differed demographically from state statistics. Respondents were younger, more educated, and poorer than state estimates; this is likely due to one platform’s strong base of university and college students and alumni in Georgia. Our survey was diverse, with 18.1% identifying with a sexual minority population. The proportion of lesbian and bisexual respondents was three times higher than general population estimates for Georgia, [40] although state-wide statistics may underestimate sexual minority membership in younger, female populations such as ours. [6, 41] Finally, our team primarily employed survey items from validated and/or published research, however not all survey items were validated (see Additional file 2); the use of validated and/or more comprehensive tools, specifically for IPV, may have better captured various types of IPV (i.e. physical, sexual, emotional, economic) experienced in our population.

## Conclusion

Though many individuals in our sample experienced hardship, minority populations were disproportionately impacted by adverse experiences in work life, home life and wellbeing. Recovery efforts in Georgia must incorporate support mechanisms for mental health and IPV, focusing particularly on the intersectional needs of racial, ethnic, and sexual minority populations. Long term impacts of pandemic-related negative experiences, such as the loss of family and friends, financial issues, career setbacks, family struggles are still unknown. Future research should be expanded to include other minority populations such as trans men or trans women, yet also studies addressing the pandemic’s impacts on men are warranted. Broad dissemination of these findings outside of peer-reviewed literature may enable individuals in Georgia, and perhaps those in other settings, to better characterize their own experiences during the pandemic and provide reassurance that they are not alone.

## Electronic supplementary material

Below is the link to the electronic supplementary material.

Additional file 1

Additional file 2

Additional flie 3

Additional file 4

## Data Availability

All data generated or analyzed during this study are included in this published article and its additional files.
