# Supplementary material for: The COVID-19 pandemic’s intersectional impact on work life, home life and wellbeing: an exploratory mixed-methods analysis of Georgia women’s experiences during the pandemic
Source: BMC Public Health. 2022 Oct 31;22:1988. doi: 10.1186/s12889-022-14285-4 (PMC9619013; doi:10.1186/s12889-022-14285-4)
Supplement: Supplementary file 1 — Additional file 1 [file 12889_2022_14285_MOESM1_ESM.docx]

**Additional file 1** Sample Advertisement on Facebook


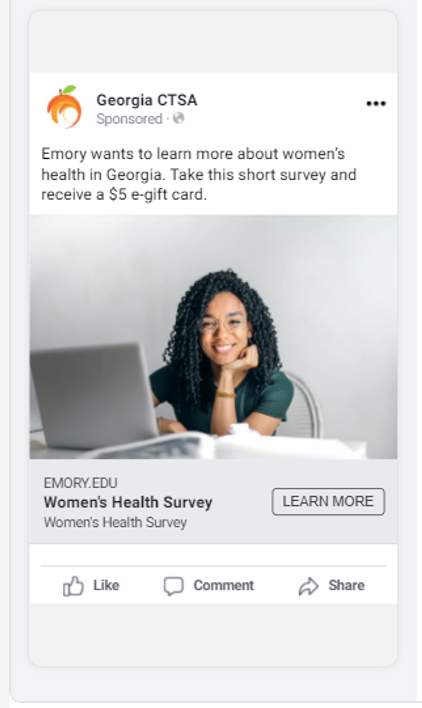


The image used in the survey promotion is a freely available image.
