# Supplementary material for: The COVID-19 pandemic’s intersectional impact on work life, home life and wellbeing: an exploratory mixed-methods analysis of Georgia women’s experiences during the pandemic
Source: BMC Public Health. 2022 Oct 31;22:1988. doi: 10.1186/s12889-022-14285-4 (PMC9619013; doi:10.1186/s12889-022-14285-4)
Supplement: Supplementary file 2 — Additional file 2 [file 12889_2022_14285_MOESM2_ESM.docx]

**Additional file 2** COVID-19 Survey

Please see references for survey domains at the end of the document.

**Demographics**

*Please tell us a little bit about yourself.*

1. How old are you?
   1. Younger than 18
   2. 18-25
   3. 26-30
   4. 31-35
   5. 36-40
   6. 41-45
   7. 46-49
   8. Older than 49
2. What gender do you identify with?
   1. Female
   2. Male
   3. Non-Binary
   4. Other (Please tell us)
   5. Decline to answer
3. What sex were you assigned at birth?
   1. Female
   2. Male
4. Have you been living in Georgia since at least January 1, 2020?
   1. Yes
   2. No

Thank you for your time! Unfortunately, this survey may not be a good fit for you at this time. If you have comments or questions, you may contact us at: [familyplanning@emory.edu](mailto:familyplanning@emory.edu).

Would you like to learn about other research projects from Emory Division of Family Planning?

1. Yes
2. No

Please enter your email address.

Please enter your phone number.

Please answer a few more questions about yourself.

1. What is your zip code?
2. What county do you live in?
3. How would you describe where you live?
   1. Rural
   2. Urban
   3. Suburban
4. How many people live in your home, including yourself?
   1. 1
   2. 2
   3. 3
   4. 4
   5. 5
   6. 6
   7. 7 or more
5. How many adults live in your home (including yourself)?
6. Who are the other adults who live in the home with you? Please check all that apply:
   1. your spouse or partner
   2. your parents
   3. your roommates
   4. your siblings
   5. your grandparents or other adult relatives not listed above
   6. other adults
7. How many children under 18 years old live in your home (any relation to you)?
8. Which of the following best describes your racial background (Check all that apply)?
   1. Alaska Native/American Indian
   2. Native Hawaiian/Other Pacific Islander
   3. Black/African American
   4. White
   5. Asian (including Southeast Asia and India)
   6. Other (Please tell us)
   7. Don’t know
   8. Decline to answer
9. Which of the following best describes your ethnicity?
   1. Hispanic or Latina/x
   2. Not Hispanic or Latina/x
   3. Decline to answer
10. What is your relationship status?
    1. Single
    2. Partnered
    3. Other (Please tell us)
    4. Decline to answer
11. Has your relationship status changed since the COVID-19 pandemic began on March 14, 2020?
    1. Yes
    2. No
12. Did you relationships status changes as a result of the COVID-19 pandemic?
    1. Yes
    2. No
13. Do you think of yourself as:
    1. Heterosexual or straight (attracted to men)
    2. Homosexual or lesbian (attracted to women)
    3. Bisexual (attracted to more than one gender)
    4. Something else
    5. Not sure
    6. Decline to answer
14. What is the highest level of education that you have completed?
    1. Less than high school
    2. High-school diploma or GED
    3. Some college or Associates degree or Technical Certification
    4. Bachelor’s degree
    5. Master’s degree/Doctoral degree
    6. Decline to answer

| Thanks for telling us a bit about yourself. The next section of this survey has several questions about how the COVID-19 pandemic has changed people's lives. For each statement below, please indicate whether the pandemic has impacted YOU or YOUR FAMILY in the way described. Content warning: Some of these questions address sensitive topics.  **Work and Employment**  Since the COVID-19 pandemic began, what has changed for you and your family? | | | |
| --- | --- | --- | --- |
| **WORK AND EMPLOYMENT** | | | |
| 1. | Laid off from job or had to close own business. |  YES |  NO |
| 2. | Reduced work hours or furloughed. |  YES |  NO |
| 3. | Had to lay-off or furlough employees or people supervised. |  YES |  NO |
| 4. | Had to continue to work even though in close contact with people who might be infected (e.g., customers, patients, co-workers). |  YES |  NO |
| 7. | Hard time doing job well because of needing to take care of people in the home. |  YES |  NO |
| 8. | Hard time making the transition to working from home. |  YES |  NO |
| 9. | Provided direct care to people with the disease (e.g., doctor, nurse, patient care assistant, radiologist). |  YES |  NO |
| 10. | Provided supportive care to people with the disease (e.g., medical support staff, custodial, administration). |  YES |  NO |
| 11. | Provided care to people who died as a result of the disease. |  YES |  NO |
| 12. | Childcare or babysitting unavailable when needed. |  YES |  NO |
| 13. | Difficulty taking care of children in the home. |  YES |  NO |
| 15. | Had to take over teaching or instructing a child. |  YES |  NO |
| 18. | Had to move or relocate. |  YES |  NO |
| 19. | Became homeless. |  YES |  NO |
| 20. | Increase in verbal arguments or physical conflict with a partner or spouse. |  YES |  NO |
| 22. | Increase in verbal arguments or physical conflict with children in home. |  YES |  NO |
|  | Increase in verbal arguments or physical conflict among children in the home. | YES | NO |
| 25. | Unable to get enough food or healthy food. |  YES |  NO |
| 27. | Unable to pay important bills like rent or utilities. |  YES |  NO |
| 28. | Difficulty getting places due to less access to public transportation or concerns about safety. |  YES |  NO |
| 29. | Unable to get needed medications (e.g., prescriptions or over-the-counter). |  YES |  NO |
| 32. | Increase in mental health problems or symptoms (e.g., mood, anxiety, stress). |  YES |  NO |
| 33. | Increase in sleep problems or poor sleep quality. |  YES |  NO |
| 34. | Increase in use of alcohol or substances. |  YES |  NO |
| 35. | Unable to access mental health treatment or therapy. |  YES |  NO |
| 38. | Increase in health problems not related to the coronavirus. |  YES |  NO |
| 39. | Less physical activity or exercise. |  YES |  NO |
| 40. | Overeating or eating more unhealthy foods (e.g., junk food). |  YES |  NO |
| 43. | Unable to access medical care for a serious condition (e.g., dialysis, chemotherapy). |  YES |  NO |
| 44. | Got less routine medical care than usual. |  YES |  NO |
| 46. | Currently have symptoms of the coronavirus but have not best tested. |  YES |  NO |
| 47. | Tested and currently have this disease. |  YES |  NO |
| 48. | Had symptoms of the coronavirus but never tested. |  YES |  NO |
| 49. | Tested positive for the coronavirus but no longer have it. |  YES |  NO |
| 50. | Got medical treatment due to severe symptoms of the coronavirus. |  YES |  NO |
| 51. | Hospital stay due to the coronavirus. |  YES |  NO |
| 53. | Death of close friend or family member from the coronavirus. |  YES |  NO |

**Health Insurance**

1. Did you have health insurance **before** the COVID-19 pandemic?
2. No
3. Yes
4. If yes, what type?
5. Public coverage - Medicaid/Medicare
6. Private coverage - Purchased on the Marketplace/Exchange
7. Private coverage - Provided by an employer
8. Other; please specify:
9. Do you currently have health insurance?
10. No, lost due to pandemic
11. No, lost due to other circumstances
12. Yes, currently have insurance
13. If yes, what type?
14. Public coverage - Medicaid/Medicare
15. Private coverage - Purchased on the Marketplace/Exchange
16. Private coverage - Provided by an employer
17. Other; please specify
18. What months did you not have health insurance?
19. January 2020
20. February 2020
21. March 2020
22. April 2020
23. May 2020
24. June 2020
25. July 2020
26. August 2020
27. September 2020
28. October 2020
29. November 2020
30. December 2020
31. Don’t know
32. Decline to answer

**Income**

- - - 1. Which of the following sources do you receive money from in a typical month? (Select all that apply)

1. My Job
2. My spouse/partner’s job
3. Child support
4. Social security or disability
5. Supplemental Nutrition Assistance Program (SNAP or food stamps)
6. Welfare of Temporary Assistance for Needy Families (TANF)
7. Aid for dependent child(ren)
8. Other family (not living with you)
9. Other family (living in household)
10. Stipend/living allowance
11. Other (Please tell us)
12. Decline to answer
13. In 2019, what was your average household monthly income before taxes?
14. $0 to $1,012
15. Between $1,012 and $2,226
16. More than $2,226
17. Don’t know/not sure
18. Decline to answer
    - - 1. In 2019, what was your average household monthly income before taxes?
19. $0 to $1,732
20. Between $1,732 and $3,810
21. Above $3,810
22. Don’t know/not sure
23. Decline to answer

In 2019, what was your average household monthly income before taxes?

1. $0 to $2,092
2. Between $2,092 and $4,602
3. Above $4,602
4. Don’t know/not sure
5. Decline to answer

In 2019, what was your average household monthly income before taxes?

1. $0 to $2,452
2. Between $2,452 and $5,394
3. Above $5,394
4. Don’t know/not sure
5. Decline to answer

In 2019, what was your average household monthly income before taxes?

1. $0 - $2,812
2. Between $2,812 and $6,186
3. Above $6,186
4. Don’t know/not sure
5. Decline to answer

In 2019, what was your average household monthly income before taxes?

1. $0 to $3,172
2. Between $3,172 and $6,978
3. Above $6,978
4. Don’t know/not sure
5. Decline to answer

In 2019, what was your average household monthly income before taxes?

1. $0 to $3,352
2. Between $3,352 and $7,770
3. Above $7,770
4. Don’t know/not sure
5. Decline to answer
6. Which statement best describes your current monthly household income?
   1. Monthly household income has NOT CHANGED since the COVID-19 pandemic started on March 14, 2020.
   2. Monthly household income is LOWER since the COVID-19 pandemic started on March 14, 2020.
   3. Monthly household income is HIGHER since the COVID-19 pandemic started on March 14, 2020.
7. Did you receive unemployment payments during the pandemic?
   1. Yes
   2. No
   3. Not applicable
      1. If no, why do you think you didn’t receive unemployment payments?
8. Did you receive a stimulus check or direct deposit from the federal government?
   1. Yes
   2. No
   3. Unsure
      1. If no, why do you think you didn’t receive stimulus payments?
9. If yes, how did you use the money? (Select all that apply)
   - 1. Groceries
     2. Rent or mortgage
     3. Car payment
     4. Utility bills
     5. Childcare
     6. Medical bills
     7. Health insurance
     8. Credit card debt
     9. School debt
     10. Savings
     11. Shopping for personal items (non-necessities)
     12. Shopping for household necessities
     13. Other (Please tell us) ___

**Reproductive Coercion** The following questions are about reproductive coercion. Reproductive coercion is when another person tries to control your reproductive choices in order to control your life.

1. Since March 14, 2020 has a partner told you not to use any birth control (like the pill, shot, ring, etc.)?
2. Yes
3. No
4. Since March 14, 2020 has a partner told you he would leave you if you did not get pregnant?
5. Yes
6. No
7. Since March 14, 2020 has a partner hurt you physically because you did not agree to get pregnant?
8. Yes
9. No
10. Since March 14, 2020 has a partner made you have sex without a condom, or damaged a condom, so you would get pregnant?
11. Yes
12. No
13. If you answered yes to any of the above questions, has this experience changed since the COVID-19 pandemic (since March 14, 2020)?
14. The situation is much worse
15. The situation is slightly worse
16. No change
17. The situation is lightly better
18. The situation is much better

**Intimate Partner Violence**

The following questions are about your past or present experiences with verbal, physical or sexual abuse from a partner.

1. Has a boyfriend or partner ever been threatening or abusive towards you, verbally or physically?
2. Yes
3. No
4. Is this your current boyfriend or partner?
5. Yes – follow does questions
6. No – follow did questions
7. Since March 14, 2020 how often did your partner physically hurt you?
8. Never
9. Rarely
10. Sometimes
11. Fairly often
12. Frequently
13. Since March 14, 2020 how often did your partner insult or talk down to you?
14. Never
15. Rarely
16. Sometimes
17. Fairly often
18. Frequently
19. Since March 14, 2020 how often did your partner threaten you with harm?
20. Never
21. Rarely
22. Sometimes
23. Fairly often
24. Frequently
25. Since March 14, 2020 how often did your partner scream or curse at you?
26. Never
27. Rarely
28. Sometimes
29. Fairly often
30. Frequently
31. If you answered yes to any of the above questions, has this experience changed since the COVID-19 pandemic (since March 14, 2020)?
32. The situation is much worse
33. The situation is slightly worse
34. No change
35. The situation is lightly better
36. The situation is much better

**Impact of the COVID-19 pandemic on you and your family**

Please describe impact that the pandemic has had on you and your family. You may type as much as you want and address any areas not mentioned above.

**Thank you for taking our survey!** Please enter your contact information below so that we can send you a **$5 gift card** for your time.

Email address:

Phone number:

**Thank you for your time.**

Please find a list of state and national resources here:

Planned Parenthood Health Educator Text “PPNOW” to 774363 (PPINFO)

National Domestic Violence Hotline <https://www.thehotline.org/help/> 1-800-799-SAFE (7233)

Georgia Department of Public Health COVID-19 Hotline 844-442-2681

Substance Abuse and Mental Health Services Administration Hotline 1-800-622-HELP

National Suicide Prevention Lifeline 1-800-273-8255

Contact us at: familyplanning@emory.edu

**Sexual and reproductive health survey topics and measures, online survey in Georgia USA (2020)**

| Survey topics | | Question type / description | Publication |
| --- | --- | --- | --- |
| A. | Basic demographics | Multiple choice questions on age, sex, gender, living situation, education, race, ethnicity | None |
| B. | Income | Multiple choice questions on income classification (high, medium, low based on persons per household), unemployment, stimulus checks | [1, 2] |
| C. | General health | Multiple choice questions on health history and health insurance | None |
| D. | Sexual activity/behavior | Multiple choice questions on frequency, partners, desire, contraceptive method at last sex | [3] |
| E. | Condom access | Multiple choice questions on condom purchasing and associated stress | None |
| F. | Contraception / birth control access | Multiple choice questions on contraceptive access, associated stress, and level of difficulty | None |
| G. | Access to medical care | Multiple choice questions on appointment type, barriers, telehealth utilization, and associated stress | None |
| H. | Reproductive coercion (RC) and intimate partner violence (IPV) | Multiple choice questions about frequency, recency, and type of RC or IPV | [4, 5] |
| I. | Pregnancy | Multiple choice questions on family planning and pregnancy options. One open-ended question about the inability to access abortion | [1] |

**Publications**

1. Clark EA, Cordes S, Lathrop E, Haddad LB. Abortion restrictions in the state of Georgia: Anticipated impact on people seeking abortion. Contraception. 2021;103:121-6.

2. U.S. Federal Poverty Guidelines Used to Determine Financial Eligibility for Certain Federal Programs. Washington DC: US Department of Human and Health Services; 2019.

3. Li W, Li G, Xin C, Wang Y, Yang S. Challenges in the Practice of Sexual Medicine in the Time of COVID-19 in China. J Sex Med. 2020;17:1225-8.

4. Kraft JM, Snead MC, Brown JL, et al. Reproductive Coercion Among African American Female Adolescents: Associations with Contraception and Sexually Transmitted Diseases. J Womens Health. 2021;30:429-37.

5. Grasso DJ, Briggs-Gowan MJ, Carter AS, Goldstein BL, Ford JD. Profiling COVID-related experiences in the United States with the Epidemic-Pandemic Impacts Inventory: Linkages to psychosocial functioning. Brain and Behavior. 2021;n/a:e02197.
