# Supplementary material for: The COVID-19 pandemic’s intersectional impact on work life, home life and wellbeing: an exploratory mixed-methods analysis of Georgia women’s experiences during the pandemic
Source: BMC Public Health. 2022 Oct 31;22:1988. doi: 10.1186/s12889-022-14285-4 (PMC9619013; doi:10.1186/s12889-022-14285-4)
Supplement: Supplementary file 3 — Additional flie 3 [file 12889_2022_14285_MOESM3_ESM.docx]

**Additional file 3** Comparison of Study Sample and State Demographic Characteristics

|  | Sample | State of GA | P value |
| --- | --- | --- | --- |
| Characteristic | (%) | (%) |  |
| Age (years) (N=423)  18-25  26-30  31-35  36-40  41-45  46-49 | 19.1  18.7  26.0  14.9  14.9  6.4 | 21.4  15.2  15.1  16.0  15.9  16.5 | <0.001 |
| Race (n=420)  White  Black or African American  Other race(s) | 59.2  29.8  11.0 | 57.8  31.9  10.3 | 0.626 |
| Ethnicity (n=417)  Non-Hispanic  Hispanic | 92.3  7.8 | 90.2  9.8 | 0.144 |
| Sexual orientation (n=410)  Heterosexual  Bisexual or Homosexual | 83.7  16.3 | 95.1  4.9 | <0.001 |
| Education level (n=422)  High school/GED/some college  Bachelor’s degree or higher | 43.4  56.6 | 67.5  32.5 | <0.001 |
| Income level  Below federal poverty line  Above federal poverty line | 31.3  68.7 | 19.5  80.5 | <0.001 |
| Residential setting (N=423)  Urban or suburban  Rural | 80.1  19.9 | 83.0  17.0 | 0.118 |
| Health insurance (n=419)  Currently have health insurance  Do not have health insurance | 84.2  15.8 | 86.6  13.4 | 0.158 |

**References for data**

**Age groups:** Data for GA, 2010 census data, females only, cited from: <http://georgia.us.censusviewer.com/client>

Date accessed: Jul 1, 2021.

**Sexual orientation**: Data for GA, year 2017, females only, ages 18 and older, cited from: <https://williamsinstitute.law.ucla.edu/visualization/lgbt-stats/?topic=LGBT&area=13&characteristic=female#about-the-data>

Date accessed: Jul 1, 2021.

**Educational level**: Data for GA, year 2019, males and females, all ages, cited from: <https://data.census.gov/cedsci/profile?g=0400000US13> Date accessed: Jul 1, 2021.

**Race**: Data for GA, year 2019, males and females, all ages, cited from: <https://data.census.gov/cedsci/profile?g=0400000US13>

Date accessed: Jul 1, 2021.

**Ethnicity**: Data for GA, year 2019, males and females, all ages, cited from: <https://data.census.gov/cedsci/profile?g=0400000US13>

Date accessed: Jul 1, 2021.

**Income level:** Data for GA, cited from: <https://dch.georgia.gov/federal-poverty-guidelines-0>

Date accessed: Jul 1, 2021.

**Residential setting**: Data for GA, year 2019, males and females, all ages, cited from: <https://data.census.gov/cedsci/profile?g=0400000US13>

Date accessed: Jul 1, 2021.

**Health insurance**: Data for GA, year 2019, males and females, all ages, cited from: <https://data.census.gov/cedsci/profile?g=0400000US13>

Date accessed: Jul 1, 2021.
