# Supplementary material for: The COVID-19 pandemic’s intersectional impact on work life, home life and wellbeing: an exploratory mixed-methods analysis of Georgia women’s experiences during the pandemic
Source: BMC Public Health. 2022 Oct 31;22:1988. doi: 10.1186/s12889-022-14285-4 (PMC9619013; doi:10.1186/s12889-022-14285-4)
Supplement: Supplementary file 4 — Additional file 4 [file 12889_2022_14285_MOESM4_ESM.docx]

| **Table 3** | | | | | | | |
| --- | --- | --- | --- | --- | --- | --- | --- |
| *4-Class Solution: Conditional Item Probabilities and Univariate Entropy Scores* | | | | | | | |
|  |  |  |  | **Classes** | | | |
|  | ***n*** | **%** | ***e*** | **1** | **2** | **3** | **4** |
| Laid off | 423 | 22.0% | .107 | 0.06 | 0.65 | 0.27 | 0.19 |
| Reduced Work Hours | 422 | 38.6% | .092 | 0.22 | 0.78 | 0.38 | 0.40 |
| Laid Off Others | 422 | 5.2% | .079 | 0.00 | 0.24 | 0.04 | 0.05 |
| Continued Working Despite Exposure | 422 | 46.0% | .062 | 0.44 | 0.64 | 0.53 | 0.37 |
| Hard Time Working Due to Caretaking | 422 | 34.6% | .199 | 0.13 | 0.67 | 0.80 | 0.12 |
| Hard Time Working from Home | 422 | 31.8% | .084 | 0.10 | 0.39 | 0.44 | 0.39 |
| Provided Direct Care to Covid+ | 422 | 10.9% | .073 | 0.09 | 0.37 | 0.06 | 0.09 |
| Provided Supportive Care to Covid+ | 421 | 13.1% | .070 | 0.07 | 0.37 | 0.13 | 0.11 |
| Provided Care to Persons who Died of Covid | 423 | 5.7% | .061 | 0.02 | 0.17 | 0.04 | 0.07 |
| Childcare Unavailable | 423 | 25.5% | .225 | 0.11 | 0.46 | 0.70 | 0.00 |
| Difficulty Taking Care of Children | 423 | 24.8% | .232 | 0.07 | 0.52 | 0.69 | 0.01 |
| Having to Take Over Teaching Children | 423 | 37.8% | .301 | 0.21 | 0.67 | 0.95 | 0.03 |
| Moved or Relocated | 422 | 12.3% | .077 | 0.01 | 0.27 | 0.14 | 0.16 |
| Homelessness* | 421 | 4.0% | - | - | - | - | - |
| Increased Conflict with Adults | 421 | 28.5% | .148 | 0.01 | 0.76 | 0.39 | 0.29 |
| Increased Conflict with Children | 423 | 36.8% | .172 | 0.01 | 0.57 | 0.44 | 0.03 |
| Increased Conflict Among Children | 423 | 36.5% | .194 | 0.02 | 0.57 | 0.46 | 0.00 |
| Difficulty Getting Food or Healthy Food | 422 | 20.1% | .140 | 0.03 | 0.78 | 0.19 | 0.17 |
| Unable to Pay Bills | 420 | 31.4% | .168 | 0.09 | 0.98 | 0.40 | 0.23 |
| Difficulty with Transportation | 423 | 22.0% | .099 | 0.03 | 0.50 | 0.24 | 0.27 |
| Unable to Get Medications | 420 | 14.5% | .132 | 0.02 | 0.70 | 0.07 | 0.13 |
| Increased Mental Health Problems | 423 | 77.3% | .179 | 0.37 | 0.96 | 0.92 | 0.94 |
| Increased Sleep Problems | 423 | 70.7% | .168 | 0.32 | 0.98 | 0.89 | 0.81 |
| Increased Alcohol/Substance Use | 422 | 28.2% | .096 | 0.08 | 0.54 | 0.27 | 0.38 |
| Barriers to Mental Health Services | 418 | 20.6% | .124 | 0.02 | 0.71 | 0.16 | 0.24 |
| Increased Physical Health Problems | 421 | 29.7% | .161 | 0.01 | 0.84 | 0.34 | 0.34 |
| Less Physical Activity | 423 | 66.9% | .108 | 0.40 | 0.91 | 0.84 | 0.69 |
| Overeating | 423 | 65.2% | .100 | 0.40 | 0.85 | 0.81 | 0.69 |
| Barriers to Healthcare | 422 | 14.2% | .123 | 0.01 | 0.64 | 0.10 | 0.14 |
| Less Routine Healthcare | 422 | 51.2% | .123 | 0.20 | 0.93 | 0.55 | 0.60 |
| Current Covid Symptoms but Not Tested* | 423 | 4.3% | - | - | - | - | - |
| Currently Test Positive for Covid* | 423 | 2.4% | - | - | - | - | - |
| Had Covid Symptoms but Never Tested | 421 | 9.5% | .095 | 0.07 | 0.41 | 0.11 | 0.01 |
| Tested Positive for Covid | 422 | 7.1% | .058 | 0.04 | 0.17 | 0.07 | 0.07 |
| Treatment for Severe Covid* | 422 | 4.5% | - | - | - | - | - |
| Hospitalization Due to Covid* | 423 | 1.9% | - | - | - | - | - |
| Death of Family/Friend Due to Covid | 422 | 17.8% | .082 | 0.03 | 0.38 | 0.17 | 0.24 |
| *Note. n* = number of participants who contributed to an item. % = base-rate among contributing respondents. *e* = univariate entropy. Darker shades correspond to higher conditional item probabilities. * Base-rate <5% and not included in the LCA. | | | | | | | |
